# Current Status of Baricitinib as a Repurposed Therapy for COVID-19

**DOI:** 10.3390/ph14070680

**Published:** 2021-07-15

**Authors:** Maha Saber-Ayad, Sarah Hammoudeh, Eman Abu-Gharbieh, Rifat Hamoudi, Hamadeh Tarazi, Taleb H. Al-Tel, Qutayba Hamid

**Affiliations:** 1 Department of Clinical Sciences, College of Medicine, University of Sharjah, Sharjah 27272, United Arab Emirates; sara-hammoudeh@hotmail.com (S.H.); rhamoudi@sharjah.ac.ae (R.H.); qalheialy@sharjah.ac.ae (Q.H.); 2 Sharjah Institute for Medical Research, University of Sharjah, Sharjah 27272, United Arab Emirates; taltal@sharjah.ac.ae; 3 Department of Medical Pharmacology, Faculty of Medicine, Cairo University, Cairo 11559, Egypt; 4 Division of Surgery and Interventional Science, University College London, London WC1E 6BT, UK; 5 Department of Medicinal Chemistry, College of Pharmacy, University of Sharjah, Sharjah 27272, United Arab Emirates; htarazi@sharjah.ac.ae; 6 Meakins-Christie Laboratories, Research Institute of the McGill University Health Center, Montreal, QC H4A 3J1, Canada

**Keywords:** bioinformatics, transcriptomics, gene expression, baricitinib, JAK inhibitors, COVID-19, clinical trial

## Abstract

The emergence of the COVID-19 pandemic has mandated the instant (re)search for potential drug candidates. In response to the unprecedented situation, it was recognized early that repurposing of available drugs in the market could timely save lives, by skipping the lengthy phases of preclinical and initial safety studies. BenevolentAI’s large knowledge graph repository of structured medical information suggested baricitinib, a Janus-associated kinase inhibitor, as a potential repurposed medicine with a dual mechanism; hindering SARS-CoV2 entry and combatting the cytokine storm; the leading cause of mortality in COVID-19. However, the recently-published Adaptive COVID-19 Treatment Trial-2 (ACTT-2) positioned baricitinib only in combination with remdesivir for treatment of a specific category of COVID-19 patients, whereas the drug is not recommended to be used alone except in clinical trials. The increased pace of data output in all life sciences fields has changed our understanding of data processing and manipulation. For the purpose of drug design, development, or repurposing, the integration of different disciplines of life sciences is highly recommended to achieve the ultimate benefit of using new technologies to mine BIG data, however, the final say remains to be concluded after the drug is used in clinical practice. This review demonstrates different bioinformatics, chemical, pharmacological, and clinical aspects of baricitinib to highlight the repurposing journey of the drug and evaluates its placement in the current guidelines for COVID-19 treatment.

## 1. Introduction

Drug repurposing is currently the most significant strategy followed in the treatment guidelines of the unprecedented COVID-19 pandemic. Due to the swift spread of the SARS-CoV2 infection all over the world, it was early recognised that repurposing of available drugs in the market could save time and effort, and more importantly, lives. With limited data available on the nature of the disease, especially at the start of the pandemic, many drugs have been suggested, including antiviral, other antimicrobials, and immunomodulators [1]. As repurposed drugs are already in clinical use, there is relative confidence in their safety, at least when used for their original purpose [2]. Moreover, drug repurposing saves the tremendous cost and lengthy time of developing a new drug that averages $2.6 billion and 10 years or more, and overcomes the complexity of new drug approvals from the licensing authorities [3]. However, repurposing dictates enrollment of patients in clinical trials for the specific new indication, in this case, treatment of COVID-19.

In the course of viral infections, an appropriate antiviral immune response requires the activation of the inflammatory pathways of the immune system. In contrast, exaggerated response of the host’s immune system can cause severe disease [4]. A subgroup of patients with severe respiratory disease due to COVID-19 may feature a “cytokine release syndrome”, or “cytokine storm” (CS), which is linked to increased activation of Janus-associated kinase (JAK) signalling cascade. During the CS, the excessive production of pro-inflammatory cytokines leads to acute lung injury that can progress into Acute Respiratory Distress Syndrome (ARDS), a major cause of mortality in COVID-19 [5,6,7,8]. Thus, JAK-inhibitors were postulated to have a useful role in treating COVID-19 patients.

This review will discuss different perspectives of barictinib repurposing in COVID-19 treatment; including bioinformatics analysis, using relevant datasets; chemistry, demonstrating assessment of the structural activity relationship; pharmacology, reporting key kinetics and clinical use of baricitinib. Finally, the outline of the completed and ongoing clinical trials on this medication will be discussed.

## 2. Literature Search Strategy

Two authors (M.S.-A. and E.A.-G.) did a comprehensive search of PubMed, EMBASE, and Cochrane Library up to 30 May 2021. They screen titles, abstracts, and full texts independently to evaluate the articles for their eligibility.

**Figure 1:**
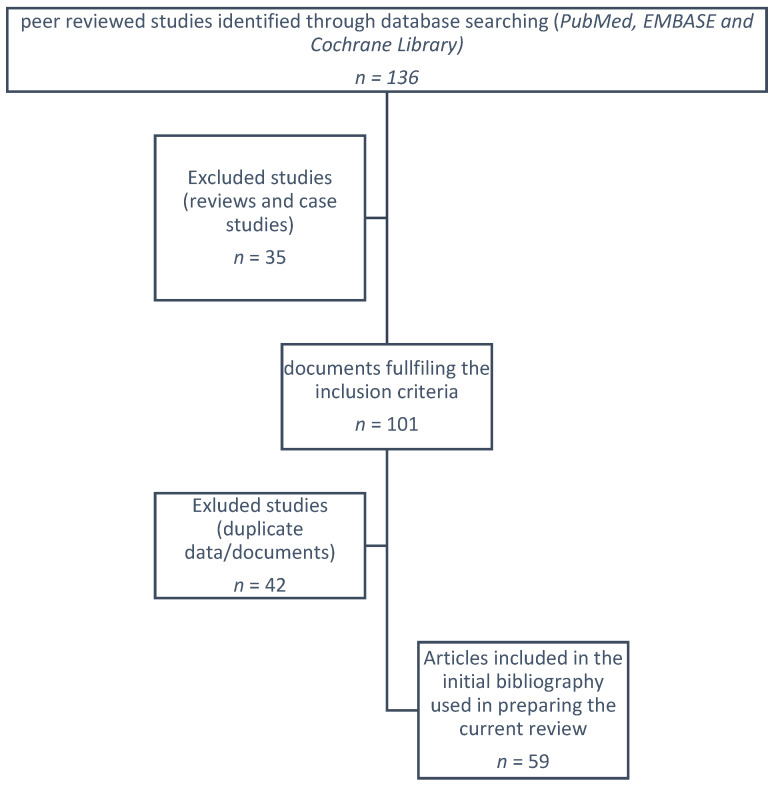
Flow chart of database searching.

The methodology of search followed the Preferred Reporting Items for Systematic Reviews and Meta-Analysis (Figure 1). The search strategy included the following relevant terms: “baricitinib AND COVID19”, “baricitinib AND COVID-19”, “baricitinib AND SARS-CoV-2”, “JAK inhibitors AND COVID-19”, “Jak inhibitors AND COVID19, “Jak inhibitors AND SARS-CoV-2”, “baricitinib AND COVID-19 AND pre-clinic” and “animal model AND SARS-CoV-2 AND baricitinib”. The inclusion criteria were English language, in-vitro, in-vivo, pre-clinical studies, molecular docking (in silico) studies, randomised control trials, prospective and retrospective cohort studies. All reviews and case studies were excluded. Unpublished clinical trials were also identified from the clinical trial registry platforms (http://clinicaltrials.gov/ accessed on 30 May 2021).

## 3. Pre-clinical Studies

### 3.1. In-Silico Studies

Using bioinformatics tools and Artificial intellegince (AI) algorithms suggested baricitinib as a potential effective therapy for COVID-19 treatment [9]. AI and machine learning have been explored in many aspects of life sciences and health care. Applications in pharmaceutical industry encampass drug design and development, manufaturing, quality control, product management, as well as clinical trial design and monitoring [10]. However, AI-driven approaches have been underutilised in drug design and development. With the unprecedented spread of the COVID-19 to become a global pandemic, quick repurposing of current medications was essential to face the global health problem. AI was suggested as an effective solution. Accordingly, several studies have been published on repurposed drugs, including baricitinib [9]. We analysed different database to demonstrate how baricitinib was recommended as a potential therapy for COVID-19 through bioinformatics.

### 3.2. Role of Bioinformatics in Unveiling New Opportunities for Drug Repurposing

Baricitinib was suggested by BenevolentAI’s large knowledge graph repository of structured medical information, including neural networks. The knowledge graph is a collection of hundreds of entities and relationships from the literature; on diseases, drugs, proteins, genes, and clinical trials. It is dynamically updated every week as a collaborative work of scientists from various backgrounds [11]. A myriad of neural networks are used in the AI approach of drug development and repurposing (Table S1 and Figure S1) [12,13,14,15,16,17,18]. The integration of available data on baricitinib suggested a promising value of the drug in the treatment of COVID-19.

### 3.3. Application of Bioinformatics on Baricitinib-Treated Models and COVID-19 Host-Related Factors

#### 3.3.1. Expression of Cytokines

We retrieved the expression profile of systemically barictinib-treated and ruxolitinib-treated C3H/HeJ grafted model of alopecia areata for 12 weeks from the datasets GSE61552 and GSE45514 deposited in Gene Expression Omnibus. We identified the differentially expressed genes using AltAnalyze Software and filtered the genes to include only those with adjusted *p*-value < 0.8 and fold change < − 2 or > 2. We analysed the differential transcriptome, using Metascape to identify the enriched functional clusters and pathways [19].

Our analysis of the baricitinib-treated samples revealed that the transcriptome suppressed by baricitinib is enriched in genes implicated in the regulation of inflammatory and humoral immune responses in response to viral and bacterial infections through the regulation of chemotaxis and migration of immune effectors such as neutrophils, eosinophils, monocytes, macrophages, granulocytes, natural killer cells, and lymphocytes (i.e., T-cells). Moreover, the suppressed transcriptome was enriched in transcripts involved in the production of and response to cytokines and chemokines, including interleukins (IL1B, IL4, IL10, IL12, IL-17, IL-13, IL23, and IL24), chemokines (CCL1, CCL2, CCL3, CCL4, CCL5, CCL17, CCL20, CCL22, CCL24, CXCL3, CXCL9, CXCL10, and CXCL11), interferon-alpha/beta/gamma, and tumour necrosis factor family members (TNF, TNFSF11, and TNFSF10). Beyond its effect on the immune response, baricitinib treatment appears to suppress tissue remodelling, ionic and transmembrane transport, as well as a cellular response to stimuli (e.g., lipopolysaccharides, lipids, biotic stimuli, and organic compounds), (details are available in the supplementary material).

Comparative analysis was performed on the ruxolitinib-treated model to identify the commonly suppressed pathways and genes. There were 481 genes widely suppressed by the two treatments (Figure S2), which are significantly implicated in immune response and defence mechanisms (Figure S3). Cytokines and chemokines that were commonly suppressed under the effect of either drug included CCL1, CCL5, CCL3, CCL17, CCL4, CCL24, CCL2, CXCL10, CXCL9, CXCL11, CXCL3, IFNG, TNFSF10, IL1B, and IL23A.

Previous studies similarly revealed the suppressive effect of baricitinib on the production and secretion of a wide range of cytokines, including CXCL9, CXCL10, and CXCL112 [20,21]. Moreover, baricitinib was proven to inhibit the differentiation of T helper cells towards pro-inflammatory phenotypes (e.g., TH1 and TH17) by inhibiting the production of IL-1β, IL-6, IL-12, and IL-23 amongst other immune mediators [22] The administration of baricitinib systemically to rheumatoid arthritis patients was shown to reduce neutrophils count [23].

#### 3.3.2. Expression of Viral Entry Receptors

**Figure 2:**
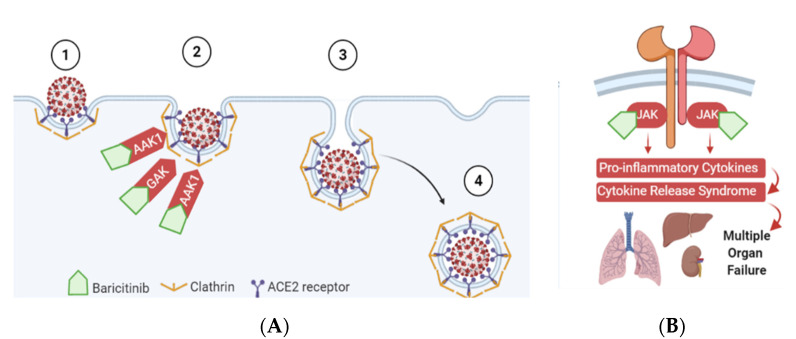
Dual mechanism of action of baracitinib; (**A**) to inhibit clathrin-mediated endocytosis of the SARS-CoV2, and (**B**) to inhibit the JAK-mediate release of pro-inflammatory cytokines.

The attachment spike glycoprotein of SARS-CoV-2 uses host cell attachment factors to mediate the viral entry, namely, angiotensin-converting enzyme 2 (ACE2) and its activator (Figure 2), the cellular protease; transmembrane protease serine 2 (TMPRSS2), [24]. Baricitinib binds to AP2-associated protein kinase 1 (AAK1) and cyclin G-associated kinase (GAK), members of the numb-associated kinase (NAK) family, which are hypothesised to facilitate viral propagation of coronavirus in epithelial cells [25]. In addition, the main mechanism of action of baricitinib as an immunomodulatory is through inhibiting JAK1/2 pathways, an effect that is favourable in slowing down the progression of CS in the context of COVID-19 [26].

**Figure 3:**
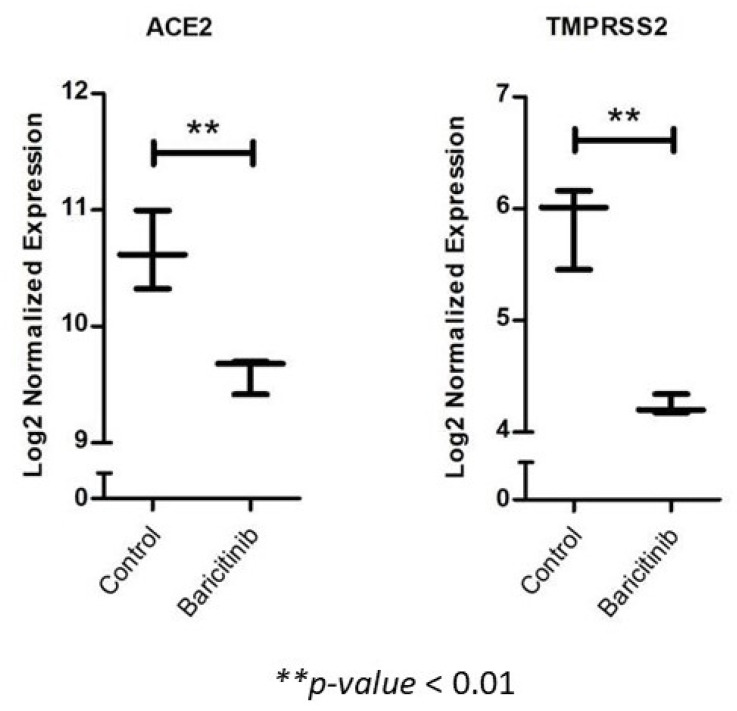
The effect of baracitinib treatment on ACE2 and TMPRSS2 expression (generated from dataset GSE61552). The dataset compiled the expression profile of systemically barictinib-treated and ruxolitinib-treated C3H/HeJ grafted model of alopecia areata. The analysis revealed a significant reduction of ACE2 and TMPRSS2 expression in the baricitinib-treated samples, compared to untreated control ones. ACE2 = angiotensin-converting enzyme 2, TMPRSS2: transmembrane protease serine 2. ** *p*-value < 0.01.

Our transcriptomic analysis of baricitinib-treated model revealed a significant downregulation of ACE2 and TMPRSS2 in patients treated with baricitinib (Figure 3).

#### 3.3.3. Structural Activity Relationship of JAK Inhibitors

**Figure 4:**
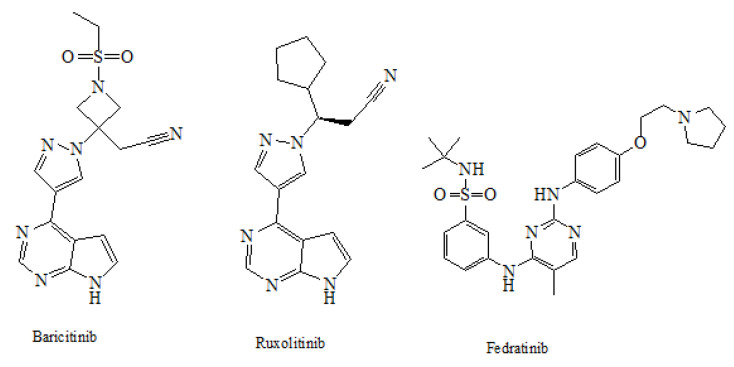
Structures of baricitinib, ruxolitinib, and fedratinib.

The currently approved JAK inhibitors include ruxolitinib, tofacitinib, baricitinib, and oclacitinib, which target JAK1/2, JAK1/3, JAK1/2, and JAK1, respectively [27,28,29]. The chemical structure of three JAK inhibitors is shown in (Figure 4).

To shed light on the structural properties and differences between these molecules which might facilitate the discovery of selective JAK inhibitors, we have calculated the physicochemical parameters (TPSA, XLOGP3, and MLOGP) of these drugs employing the SwissADME web server [30], to evaluate some of their pharmacokinetics and drug-likeness attributes. Other parameters related to absorption, distribution, elimination, and toxicity were also calculated using the PreADMET webserver (https://preadmet.bmdrc.kr/ accessed on 25 April 2021). The prediction of ADME/Tox parameters is achieved based-on well-validated QSAR models [31]. According to the calculated data given in Table 1, the physicochemical parameters of the investigated compounds demonstrate a lipophilic nature of fedratinib, whereas ruxolitinib exhibited medium lipophilicity, while baricitinib showed the most hydrophilic nature among other JAK inhibitors. Fedratinib and ruxolitinib showed higher membrane permeation compared to baricitinib. Furthermore, all of the investigated compounds showed high intestinal absorption, blood bioavailability, and plasma protein binding capabilities. The toxicity related parameter (hERG-blocking activity) suggested an increased risk for fedratinib compared to the others. Additionally, ruxolitinib and baricitinib exhibited a high probability of being P-glycoprotein modifiers, with potential drug–drug interactions.

**Figure 5:**
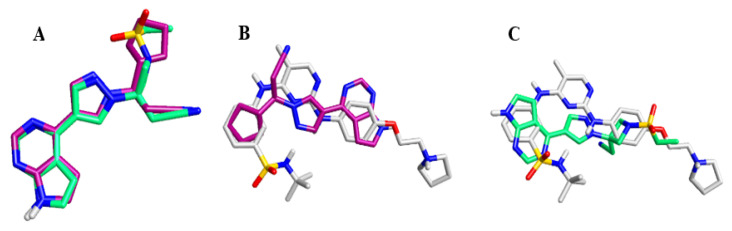
(**A**) An overlay of ruxolitinib (Purple) on baricitinib (Green); electronic similarity percentage = 82.5%. (**B**) An overlay of fedratinib (Gray) on ruxolitinib (Purple); electronic similarity percentage = 54.0%. (**C**) An overlay of fedratinib (Gray) on baricitinib (Green); electronic similarity percentage = 49.1%. Similarity and alignments were measured based on molecular fields descriptors generated by Cresset’s FieldAlign Software (version 1.0.2), [37].

Structural overlay between these molecules indicated that the similarity between ruxolitinib and baricitinib is 82.5%, whereas fedratinib highly deviates from ruxolitinib and baricitinib. Similarity and alignments were measured based on molecular fields descriptors generated by Cresset’s FieldAlign Software (version 1.0.2), (Figure 5), [37]. This theoretical finding indicates that these two molecules might share similar pharmacological and adverse effects. However, the structure of fedratinib suggests that it should have a different pharmacological profile compared to those of ruxolitinib and baricitinib. Some theoretical findings are validated through the reported biological activities associated with these drugs (Table 1), [37,38,39,40,41]. In conclusion, both ruxolitinib and baracitinib may represent promising options for COVID-19 treatment; however, the clinically reported adverse effects of these drugs and our theoretical calculations, especially for fedratinib, raise an alarming concern regarding their safety. Such drugs should be cautiously used in the clinical settings to treat COVID-19 patients.

### 3.4. In-Vitro Studies on Baricitinib in COVID-19 Models

Several studies have predicted a favourable effect of baricitinib on the immune response in COVID-19. The first key study was published by Stebbing et al., 2020 who showed that baricitinib inhibited signalling of COVID-19-related cytokines in an in-vitro model. They measured the affinities for AAK1, BIKE, and GAK, members of the human numb-associated kinase (hNAK). Baricitinib reduced viral infectivity in the human primary liver spheroid model. The effect was detected at clinically-relevant levels of exposure of baricitinib [38].

Petrone et al. assessed the effect of baricitinib on the release of IFN-γ and on a panel of soluble factors by multiplex-technology after stimulating whole-blood from COVID-19 patients with SARS-CoV-2 antigens. In mild to moderate cases, baricitinib suppressed the spike protein-specific-response, as demonstrated by significant decrease in a myriad of cytokines including IFN-γ, IL-17, IL-1β, IL-6, TNF-α, IL-4, IL-13, IL-1ra, IL-10, GM-CSF, FGF, IP-10, MCP-1, and MIP-1β [39].

Through exploring the mechanistic aspects of baracitinib effect, Stebbing et al. demonstrated first that ACE2 expression is increased by more than fivefold under the effect of interferon-α2 in a human liver cell model. Evaluating the gene differentially expression revealed that the gene response signatures associated with platelet activation were completely inhibited by the administration of baricitinib through its rapid inhibition of hNAK, a mechanism that is unique among antiviral agents [40].

### 3.5. In-Vivo Studies

Baricitinib has swiftly moved from in-silico studies to clinical trials (Table 2). There are only a few animal studies on the drug in COVID-19 treatment, the most reliable of which is the study by Hoang et al., 2020. In a non-human-primate animal model of SARS-CoV2 infection, an 8-day baricitinib treatment of adult rhesus macaques markedly and rapidly reduced macrophage production of cytokines and chemokines in the lung. In addition, treated animals had reduced neutrophil extracellular trap activity and showed limited lung pathology [41].

## 4. Repurposed Immunomodulators in Treatment of COVID-19

Aiming at a favourable clinical outcome in COVID-19 patients with CS, treatment should be initiated as early as possible. Many medications combating cytokine release and/function have been proposed for treating CS. Anakinra, an IL-1 receptor antagonist, used in rheumatoid arthritis, was proven to be helpful in cytophagic histiocytic panniculitis with secondary hemophagocytic lymphohistiocytosis, a disease associated with severe CS [43]. Tocilizumab is a blocker of the recombinant humanised IL-6 receptor that inhibits IL-6 signalling. Tocilizumab is used in the treatment of rheumatoid arthritis, juvenile idiopathic arthritis, giant cell arteritis, and has shown a promising effect in the treatment of CS complicating CAR-T cell therapy for haematological malignancies [44]. In addition, downstream inhibitors of cytokines, e.g., JAK inhibitors, are also being explored in treating CS.

## 5. Pharmacology of Baricitinib

Baricitinib was approved in 2018 by the Food and Drug Administration (FDA) and the European Medicines Agency (EMA) for the treatment of adult patients with rheumatoid arthritis (RA), as an oral disease-modifying antirheumatic medication (DMARD). It also indicated to reduce inflammation and pruritus in moderate to severe atopic dermatitis [45]. Moreover, the drug is also under clinical trials for many autoimmune diseases, including systemic lupus erythematosus [46], (NCT02708095), atopic dermatitis, juvenile idiopathic arthritis (NCT04088396 and NCT04088409), alopecia areata (NCT03570749), [47], and chronic atypical neutrophilic dermatosis (NCT04517253).

When given orally, the plasma baricitinib concentration peaks within 1.5 h and declines in a bi-exponential fashion. Baricitinib has linear and invariant pharmacokinetics, with a clearance of 17 L/h (Jack G Shi, 2014). No dose adjustment is required in patients with mild to moderate liver impairment. The drug was not studied in patients with severe liver impairment. The drug is not recommended in patients with an estimated glomerular filtration rate (eGFR) below 60 mL/min/1.73 m^2^ [48,49].

Significant adverse effects of baricitinib include serious infections, malignancies, and thrombosis. Baricitinib may lead to a variety of haematological adverse effects, including pancytopenia. Elevated liver enzymes and disturbed lipid profiles may also be observed in patients on baricitinib.

## 6. Baricitinib for COVID-19 Treatment

Supported by an AI-driven approach, several clinical trials were launched for the use of baricitinib in patients with COVID-19 (Table 2).

Baricitinib is distinguished from other JAK inhibitors by its dual action on both viral infectivity as well as on immunomodulation (Figure 2). The attachment spike glycoprotein of SARS-CoV-2 uses host cell attachment factors to mediate the viral entry, namely, angiotensin-converting enzyme 2 (ACE2) and its activator (Figure 2), the cellular protease; transmembrane protease serine 2 (TMPRSS2) [24]. Baricitinib binds to AAK1 and GAK, members of the numb-associated kinase (NAK) family which are hypothesised to facilitate viral propagation of coronavirus in epithelial cells [25]. In addition, the main mechanism of action of baricitinib as an immunomodulatory is through inhibiting JAK1/2 pathways, an effect that is favourable in slowing down the progression of CS in the context of COVID-19 as previously described (Table S2).

Several studies reported the effectiveness of baricitinib in preventing the cytokine storms that are often the cause of death for patients with COVID-19. Baricitinib was reported, in a small study on 20 patients, to prevent the progression towards a severe form of COVID-19 by modulating the patients’ immune response, leading to a favourable clinical outcome. A significant reduction of IL-1ᵦ, IL-6, and TNF-ɑ plasma levels was noted in patients treated with baracitinib [50].

Baricitinib is the only JAK inhibitor approved for COVID-19 treatment in combination with the antiviral remdesivir (ACTT-2), [42]. However, the drug has only been used in Europe in clinical trials for COVID-19 treatment so far (not recommended by NHS or other European guidelines for COVID-19 treatment).

In one of the studies carried by the Hospital of Prato in Italy, 113 patients treated with baricitinib were compared with a control group of 78. Deaths were significantly lower; none of the patients on baricitinib died, compared with five of the control group [51]. In addition, when compared to corticosteroids alone, a combination of baricitinib and corticosteroids was linked with higher improvement in pulmonary function in patients with moderate to severe SARS-CoV-2 pneumonia [52]. Currently, COV-BARRIER study has been completed, but not yet published. The study evaluates the effectiveness of baricitinib in adults hospitalised due to COVID-19 infection, (NCT04421027). The initial results show that the disease progression was not significantly reduced, but the treatment with baricitinib in addition to standard of care (including dexamethasone) significantly reduced mortality, in hospitalised patients with COVID-19.

In ACTT-2 study, the time to recovery was measured as a primary outcome for over 1000 patients who were randomised to receive remdesivir (≤ 10 days) and either baricitinib (≤ 14 days) or placebo (control). The key secondary outcome was clinical status on day 15 [42]. The study concluded that baricitinib plus remdesivir was superior to remdesivir alone, reducing the recovery time among patients with Covid-19 (10 days in the combination therapy group, compared to 18 days in the control group), more significantly among those receiving high-flow oxygen or noninvasive ventilation, with fewer serious adverse events in the combination therapy group [53] (Table 3).

## 7. Discussion and Conclusions

In response to the unprecedented COVID-19 pandemic, a plethora of medications has been repurposed in an attempt to combat the infection and its sequelae. Baricitinib was identified as a potential repurposed medication for COVID-19 treatment through multi-disciplinary fields including bioinformatics, medicinal chemistry, and pharmacology.

Previous studies showed promising dual effects of baricitinib through reducing the entry of the virus and combating the CS. Interestingly, our transcriptomic analysis of baricitinib-treated model revealed a significant downregulation of ACE2 and TMPRSS2 in patients treated with baricitinib. Moreover, through binding to numb-associated kinases, baricitinib further inhibits AAK1 and GAK-mediated endocytosis of the virus-ACE2 complex, eventually leading to a reduction in the viral load [54].

In COVID-19, the CS represents a major turn in the disease progress that contributes to complications and mortality. The immunomodulatory and anti-inflammatory effects of baricitinib support its capacity as a potential therapeutic approach for COVID19, as supported by our analysis. For instance, one of the pathogenic mechanisms of COVID19 is the uncontrolled dysregulation production of cytokines (e.g., CXCL9, CXCL10, CCL3, CCL5, IL-1β, IL-4, IL-12, IL-13, IL-17, IFNG, and TNF-α), resulting in the development of a cytokine storm, systemic inflammation, and consequently multi-organ failure [55,56]. Through the process of the development of the cytokine storm, various immune effectors, including neutrophils, monocytes, and macrophages, are recruited and activated to produce cytokines and contribute to the exacerbation of systemic inflammation [56]. Therefore, the capacity of baricitinib to suppress the production of these cytokines and subsequently suppress the recruitment of the immune effectors proved to significantly contribute to the recovery of COVID19 patients (i.e., reduced sera cytokine levels, recovery of lymphocytes, and reduced need for oxygen flow) [50].

Noteworthy, as a consequence of the exacerbated immune response that leads to the CS in COVID-19, tissue remodelling and fibrotic changes were proposed to be induced in SARS-CoV-2 infected tissues [57]. Therefore, as revealed by our analysis, the suppressive effect of baricitinib on tissue remodelling, cytoskeleton reorganisation, and extracellular matrix remodelling can potentially counteract these pathological fibrotic changes and loss of tissue function.

The structural overlay of three JAK inhibitors revealed that ruxolitinib and baricitinib are markedly identical, but fedratinib differs greatly from both. Intringuingly, the clinically documented side effects of these medicines, as well as our theoretical calculations, raise serious concerns about their safety, particularly for fedratinib. In clinical settings, such medicines should be used with close monitoring of patients for potential adverse effects, particularly disturbance of cardiac rhythm.

Currently, the only JAK inhibitor authorized for COVID-19 therapy in combination with the antiviral remdesivir (ACTT-2) is baricitinib [50]. However, other JAK inhibitors have been previously investigated through clinical trials, e.g., ruxolitinib (NCT04359290 and NCT04362137). Interestingly, fedratinib has been approved by the FDA for the treatment of Myelofibrosis in August 2019, in contrast to ruxolitinib that has been the first JAK inhibitor to be approved by the FDA in 2011 [58]. Being relatively recent in clinical practice made fedratinib a less attractive choice to try for treatment of COVID-19 [59]. Ruxolitinib and tofacitinib have off-target kinase interactions, which can lead to unwanted side effects [27,60]. For instance, it has been reported that ruxolitinib causes thrombocytopenia, anaemia, and immunosuppression [29], while tofacitinib causes anaemia and neutropenia [27]. Moreover, the pharmacokinetic properties of ruxolitinib showed that the unbound plasma concentration needed to combat the clathrin-mediated endocytosis of SARS-CoV2 is higher than the tolerated therapeutic dose. Thus, the drug may reduce the host inflammatory response without affecting the viral infectivity at therapeutic doses. Ruxolitinib has been shown to reduce cytokine levels and improve outcomes in clinical practice [61]. The drug was previously explored to manage COVID-19 (e.g., NCT04348071, NCT04355793, NCT04354714, NCT0437720). However, the studies were either withdrawn, temporarily not available, withdrawn or terminated, respectively. Intriguingly, the COVID-19 Treatment Guidelines of the National Institutes of Health recommend against the use of JAK inhibitors other than baricitinib for the treatment of COVID-19, except in clinical trials. Additionally, the use of baricitnib as a monotherapy is not recommended, except in clinical trials [53].

In comparison to other JAK inhibitors, the favourable pharmacokinetic properties of barcitinib, such as low plasma protein binding affinity, minimal interaction with cytochrome enzymes, and drug transporters, give a great chance for potential combination therapy of baricitinib with other medications [54]. Recently, a meta-analysis of eleven studies including 2367 individuals was published to evaluate the safety and effectiveness of ruxolitinib and baricitinib in patients with COVID-19. Both medications decreased the use of invasive mechanical ventilation, while having only marginal impacts on ICU admission rates and ARDS and having no effect on the length of hospitalization. The risk of mortality was reduced, most notably with the use of baricitinib [62].

Despite the fact that baricitinib was suggested by AI algorithms through integration of several database, the clinical trials on baricitinib as monotherapy for COVID-19 failed to show significant impact on the disease progression, as did the clinical trials on other JAK inhibitors. Only when used in combination with the antiviral remdesivir did baricitinib show favorable effect in hospitalized patients with SARS-CoV2 infection. The drug has been positioned in the NIH guidelines for COVID-19 in combination with remdesevir, based on ACTT-2 study, and probably with “standard of care” including dexamethasone. On the other hand, baricitinib is not recommended outside clinical trials in Europe, considering the published national guidelines of several European countries.

In conclusion, baricitinib’s role in clinical practice for the treatment of COVID-19 is still to be further elucidated. Awaiting more evidence, specific guidelines have placed baricitinib only in combination with other medications in treatment regimens of a specific category of hospitalized patients with COVID-19.

## Figures and Tables

**Table 1:** Calculated physicochemical parameters (TPSA, XLOGP3, and MLOGP) of Baricitinib, Ruxolitinib, and Fedratinib.

Properties/Drug	Baricitinib [32]	Ruxolitinib [33]	Fedratinib [34]
TPSA ^a^ (Å^2^)	128.94	83.18	116.86
Log *P*_o/w_ (XLOGP3) ^b^	−0.46	2.12	4.76
Log *P*_o/w_ (MLOGP) ^c^	−0.58	1.36	2.47
HBD ^d^	1	1	3
HBA ^e^	7	4	9
BBB ^d^	0.009148 (CNS −ve)	0.148161 (CNS −ve)	0.781302 (CNS −ve)
Caco-2 ^e^ (nm/s)	1.55292 (Low)	13.7647 (Medium)	20.9596 (Medium)
HIA ^f^	93.89 %	92.38%	93.98%
P-gp inhibitor	−ve	+ve	−ve
P-gp substrate	+ve	+ve	−ve
PPB (%)	87.49% (50%)	84.28% (97%)	83.95% (92%)
Log *K*_p_ (skin) ^g^	−3.93772	−3.97931	−2.17719
F (10%) score ^h^	0.55 (0.79)	0.55 (0.95)	0.55 (0.96)
hERG-block activity	Medium-risk	Medium-risk	High-risk

^a^: Topological polar surface area calculated as per Ertl et al. (2000), [35]. ^b^: Log *P*_o/w_ atomistic and knowledge base method calculated by XLOGP program, version 3.2.2. ^c^: Partition coefficient, Log *P*_o/w_ topological method calculated according to Moriguchi et al. (1994), [36]. ^d^: In-vivo blood–brain barrier penetration (C.brain/C.blood). ^e^: Caco2 cell permeability (nm/s). ^f^: Human Intestinal Absorption as percentage. ^g^: Skin permeation in cm/h. ^h^: Abbott Probability of more than 10% bioavailability. Values between brackets are experimentally determined and extracted from DrugBank (FDA Approved Drug Products: JAKAFI (ruxolitinib) tablets, for oral use). PPB: plasma-protein binging, F: oral bioavailability.

**Table 2:** Clinical trials on baricitinib as a therapy for COVID-19.

Repurposed JAK Inhibitor	Study Title	Study Design	Phase	Status	Clinical Trial ID
Baricitinib	Treatment of Moderate to Severe Coronavirus Disease (COVID-19) in Hospitalised Patients	Non-randomised Parallel Assignment	Phase 2	recruiting	NCT04321993
Baricitinib	A Study of Baricitinib (LY3009104) in Participants With COVID-19 (COV-BARRIER)	Randomised Parallel Assignment Double blind	Phase 3	recruiting	NCT04421027
Baricitinib	Baricitinib Therapy in COVID-19	Non-randomised Cross-over assignment	Phase 2 and 3	Completed	NCT04358614
Baricitinib (+Hydroxy chloroquine)	Baricitinib, Placebo and Antiviral Therapy for the Treatment of Patients With Moderate and Severe COVID-19	Randomised Parallel Assignment Double blind	Phase 2	recruiting	NCT04373044
Baricitinib And remdesivir ACTT-2	Adaptive COVID-19 Treatment Trial 2 (ACTT-2)	Interventional (Clinical Trial) Randomized Parallel Assignment	Phase 3	Completed	NCT04401579 Completed and published [42]
ACTT-4 Baricitinib in comparison to Remdesivir, and dexamethasone as monotherapies	Adaptive COVID-19 Treatment Trial 4 (ACTT-4)	Interventional (Clinical Trial) Randomized Parallel Assignment	Phase 3	This study closed because neither treatment regimen was significantly better than the other.	NCT04640168

**Table 3:** Summary of COVID-19 Treatment Guidelines Panel recommendations (modified from the NIH guidelines) [53].

The panel recommends the use of baricitinib combined with remdesivir for the treatment of COVID-19 in hospitalised, non-intubated patients who require oxygen supplementation (BIIa).
For the treatment of COVID-19 in hospitalized patients on high-flow oxygen or noninvasive ventilation who have evidence of clinical progression or increased markers of inflammation, the panel recommends using either baricitinib (BIIa) or tocilizumab (BIIa) in combination with dexamethasone alone or dexamethasone plus remdesivir *.
The panel discourages the use of baricitinib as a single therapy, except in a clinical trial (AIII).
The Panel recommends against the use of JAK inhibitors, except for baricitinib, to treat COVID-19 (AIII).

* Awaiting the publication of COV-BARRIER study (NCT04421027).

## Data Availability

Data sharing not applicable.

## Supplementary Materials

The following are available online at https://www.mdpi.com/article/10.3390/ph14070680/s1, Figure S1: Types of common neural networks used in machine learning for drug discovery and repurposing. A. Feed Forward (FF) Neural Network. B. Deep Convolutional Network (DCN). C. Recurrent Neural Network (RNN). Figure S2: 481 genes were commonly suppressed by baricitinib and ruxolitinib in the C3H/HeJ grafted mouse model of alopecia areata. Figure S3: The commonly suppressed transcriptome was enriched in the following functional clusters and pathways: analysed using Metascape), (Zhou et al., 2019)., Table S1: Main Neural networks used in drug design and repurposing., Table S2: Immune cells chemotaxis and migration-related functional clusters and pathways enriched in the downregulated transcriptome in baricitinib treated samples.
