# Supplementary material for: Current Status of Baricitinib as a Repurposed Therapy for COVID-19
Source: Pharmaceuticals (Basel). 2021 Jul 15;14(7):680. doi: 10.3390/ph14070680 (PMC8308612; doi:10.3390/ph14070680)
Supplement: Supplementary file 1 [file pharmaceuticals-14-00680-s001.zip › Table S1&S2.pdf]

Table S1. Main Neural networks used in drug design and repurposing.

Neural Network	Description	Uses
Perceptron/ single-layer neural network.	-The perceptron contains only two layers; input and output. There are no "hidden layers". -Developed by Frank Rosenblatt [17].	
Feed-forward neural network (FFN)	-Simple input and output layers. -Nodes do not form a cycle. -Hidden layers have no connection to the outer world. -Each perceptron in one layer is connected with each node in the next layer. -All the nodes are fully connected. -There are no back-loops. -The error of prediction is minimum (Figure S1.A).	FNNs are used in grouping drugs based on the transcriptomic profile [11].
Deep Convolutional Network (DCN)	-Neural networks used primarily for image recognition and grouping. -A Kernel is a filter used in convolutional layers to extract features. -creates unsupervised hierarchical image representations and add more complex features to it so that it can perform the task with better accuracy (Figure S1.B). -Limitations include slow computational speed and inability to consider the future input or to remember old state.	Used for the analysis of chemical images to recognize drug action at the molecular level [13]. Extracted information by CNNs can predict the binding affinity of drugs to target proteins [12].
Recurrent Neural Network (RNN)	-A modification to feed-forward (FF) networks (Quax and van Gerven, 2018). -Their architecture is temporal, with each neuron in hidden layers receives an input with a specific time lag (Figure S1.C). -Access previous information in current repetitions. Can process inputs and share any lengths and weights across time. -The computations in this model recognize/consider the previous information.	Used to generate libraries of molecules that can be then tested for a particular pharmacological action, and to predict drug-target interaction [14]. A hybrid model of CNN and RNN (molecular transformer drug target interaction-used to repurpose antivirals for COVID-19), [15]

	-The model size does not increase with the size of the input.	
Graph Representatio n Learning	-Ensures proper integration of diverse data of disease, small molecules, existing drugs, and targeted proteins, with the possible prediction of new links between known and new disease entities. Such similarities can lead to identifying drugs likely to be effective when repurposed. -The major limitation is scalability, as millions of entities in a medical knowledge graph could exist, surpassing the capacity of most existing machine learning systems.	BenevolentAI's knowledge graph has been used to identify baricitinib as a potential treatment of COVID-19 [16], (refer to the text).

Table S2. Immune cells chemotaxis and migration-related functional clusters and pathways enriched in the downregulated transcriptome in baricitinib treated samples.

Description	Term	Log10 p-value	Log10 q-value	InTerm_InList	Genes
chemokine-mediated signaling pathway	GO:0070098	−8.90	−6.25	20/88	CCR1,CCR5,EDN1,FOXC1,GPR35,CXCL3,CXCL10,CXCL9,CCL1,CCL2,CCL3,CCL4,CCL5,CCL17,CCL20,CCL22,CCL24,CXCL11,XCL1,CXCR6
neutrophil chemotaxis	GO:0030593	−8.65	−6.04	21/100	EDN1,EDN2,CXCL3,IL1B,CXCL10,CXCL9,RAC2,S100A8,CCL1,CCL2,CCL3,CCL4,CCL5,CCL17,CCL20,CCL22,CCL24,CXCL11,XCL1,DAPK2,IL23A
lymphocyte activation	GO:0046649	−8.12	−5.58	69/748	AIRE,BCL3,BST2,CASP3,CD3D,CD6,CD8A,CD8B,CD40,CTPS1,EPHB1,FANCD2,FGR,FYN,HELLS,HLA-A,IFNG,IL1B,IL2RA,IL4R,IL13,IDO1,INHA,INHBA,IRF1,ITGAL,ITK,LCK,PDCD1,PLCG2,RAC2,RORA,CCL2,CCL5,XCL1,SHH,SLAMF1,SPN,UNG,NSD2,ZAP70,FZD5,FOXN1,TNFSF11,IL18R1,PGLYRP1,EXO1,IL27RA,SOX13,SOCS5,RNF41,IKZF3,PTPN22,ICOS,LEF1,IL23A,PAG1,SLAMF7,CLEC7A,RASAL3,VTCN1,PDCD1LG2,NFKBID,HAVCR2,PGLYRP2,SLAMF6,NLRC3,TXLNA,TBC1D10C,CTSC,IL13RA2,NECTIN2,CCL3,NR1D1,BMP7,HAS2,S100A8,TNF,SKAP1,ALOX15,KIF26B,ADAMTS18,PCDH8,B4GALNT2,EPHA1,ITGA3,ITGAV,NEDD9,RET,ADGRG1,DOCK5,TNC,MMP12,PLXNB1,PML,CYTIP,SLK,NUAK1,OCSTAMP
granulocyte chemotaxis	GO:0071621	−7.24	−4.81	21/119	EDN1,EDN2,CXCL3,IL1B,CXCL10,CXCL9,RAC2,S100A8,CCL1,CCL2,CCL3,CCL4,CCL5,CCL17,CCL20,CCL22,CCL24,CXCL11,XCL1,DAPK2,IL23A
leukocyte chemotaxis	GO:0030595	−7.20	−4.78	30/223	CHGA,CCR1,CCR5,EDN1,EDN2,F7,FLT1,CXCL3,IL1B,CXCL10,CXCL9,PGF,RAC2,S100A8,CCL1,CCL2,CCL3,CCL4,CCL5,CCL17,CCL20,CCL22,CCL24,CXCL11,XCL1,PLA2G7,TNFSF11,CYP7B1,DAPK2,IL23A
chemotaxis	GO:0006935	−7.17	−4.75	60/649	ALCAM,BMP7,CHGA,CCR1,CCR5,DEFB4A,EDN1,EDN2,EPHA1,EPHA4,EPHB1,F7,FLT1,FYN,CXCL3,NRG1,IL1B,CXCL10,ITGAV,CXCL9,PGF,PLXNB1,RAC2,RET,S100A8,CCL1,CCL2,CCL3,CCL4,CCL5,CCL17,CCL20,CCL22,CCL24,CXCL11,XCL1,SHH,SPN,CNTN2,PLA2G7,FOSL1,SEMA7A,TNFSF11,ALKBH1,ARTN,PDLIM7,CYP7B1,TUBB3,CXCR6,DAPK2,FLRT3,LEF1,IL23A,ENAH,VANGL2,DSCAML1,SEMA6D,CMTM8,UNC5B,DEFB103A

T cell activation	GO:0042110	−7.07	−4.68	48/472	AIRE,BCL3,CASP3,CD3D,CD6,CD8A,CD8B,CTPS1,FANCD2,FYN,HLA-A,IFNG,IL1B,IL2RA,IL4R,IDO1,IRF1,ITGAL,ITK,LCK,PDCD1,RAC2,RORA,CCL2,CCL5,XCL1,SHH,SPN,ZAP70,FZD5,FOXN1,TNFSF11,IL18R1,SOX13,SOCS5,PTPN22,ICOS,LEF1,IL23A,PAG1,CLEC7A,RASAL3,VTCN1,PDCD1LG2,NFKBID,HAVCR2,SLAMF6,NLRC3
lymphocyte migration	GO:0072676	−6.75	−4.39	20/116	AIRE,CXCL10,ITGAL,RET,CCL1,CCL2,CCL3,CCL4,CCL5,CCL17,CCL20,CCL22,CCL24,CXCL11,XCL1,SPN,ZAP70,ARTN,CYP7B1,IL27RA
monocyte chemotaxis	GO:0002548	−6.75	−4.39	15/67	CCR1,FLT1,CXCL10,CCL1,CCL2,CCL3,CCL4,CCL5,CCL17,CCL20,CCL22,CCL24,XCL1,PLA2G7,TNFSF11
cell chemotaxis	GO:0060326	−6.58	−4.25	35/304	CHGA,CCR1,CCR5,DEFB4A,EDN1,EDN2,EPHB1,F7,FLT1,CXCL3,IL1B,CXCL10,CXCL9,PGF,RAC2,S100A8,CCL1,CCL2,CCL3,CCL4,CCL5,CCL17,CCL20,CCL22,CCL24,CXCL11,XCL1,PLA2G7,TNFSF11,CYP7B1,CXCR6,DAPK2,LEF1,IL23A,DEFB103A
Th17 cell differentiation	hsa04659	−.05	−1.45	13/107	CD3D,IFNG,IL1B,IL2RA,IL2RB,IL4R,LCK,NFKBIE,RORA,STAT1,ZAP70,IL27RA,IL23A
Th1 and Th2 cell differentiation	hsa04658	−2.61	−1.13	11/92	CD3D,IFNG,IL2RA,IL2RB,IL4R,IL13,LCK,NFKBIE,STAT1,STAT4,ZAP70

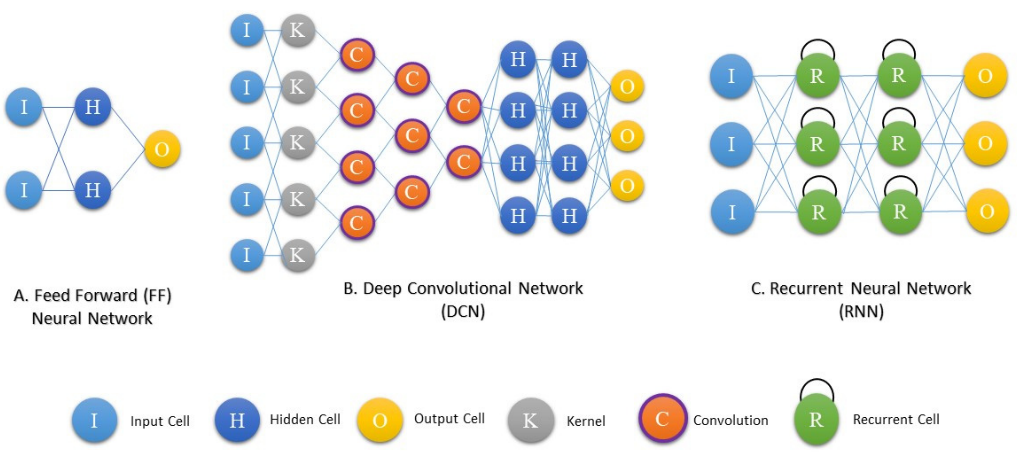

Figure S1. Types of common neural networks used in machine learning for drug discovery and repurposing. A. Feed Forward (FF) Neural Network. B. Deep Convolutional Network (DCN). C. Recurrent Neural Network (RNN).

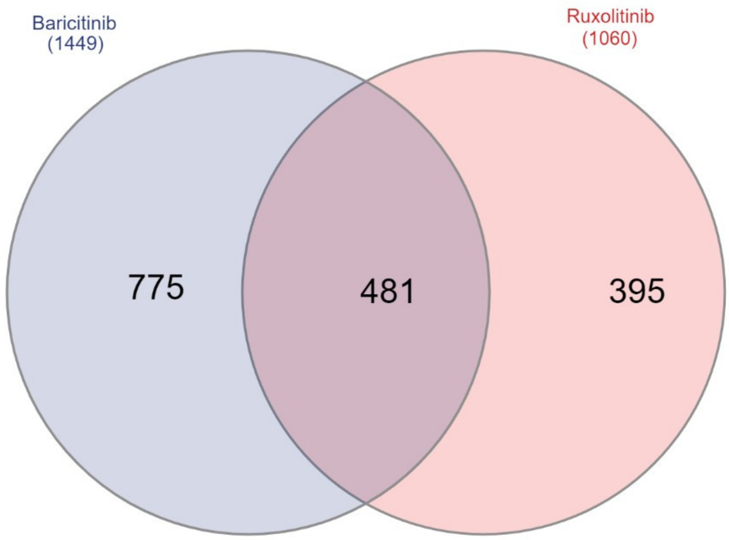

Figure S2. 481 genes were commonly suppressed by baricitinib and ruxolitinib in the C3H/HeJ grafted mouse model of alopecia areata.

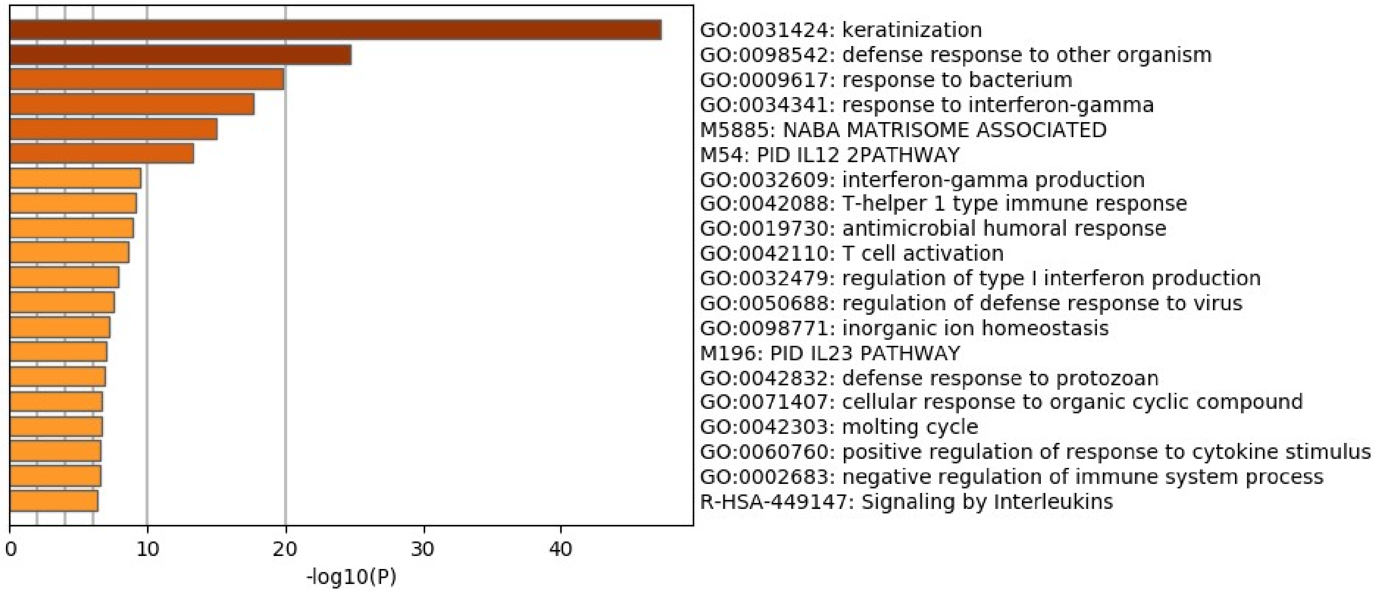

Figure S3. The commonly suppressed transcriptome was enriched in the following functional clusters and pathways: analysed using Metascape), (Zhou et al., 2019).
