# Supplementary figures and images for: Current Status of Baricitinib as a Repurposed Therapy for COVID-19
Source: Pharmaceuticals (Basel). 2021 Jul 15;14(7):680. doi: 10.3390/ph14070680 (PMC8308612; doi:10.3390/ph14070680)

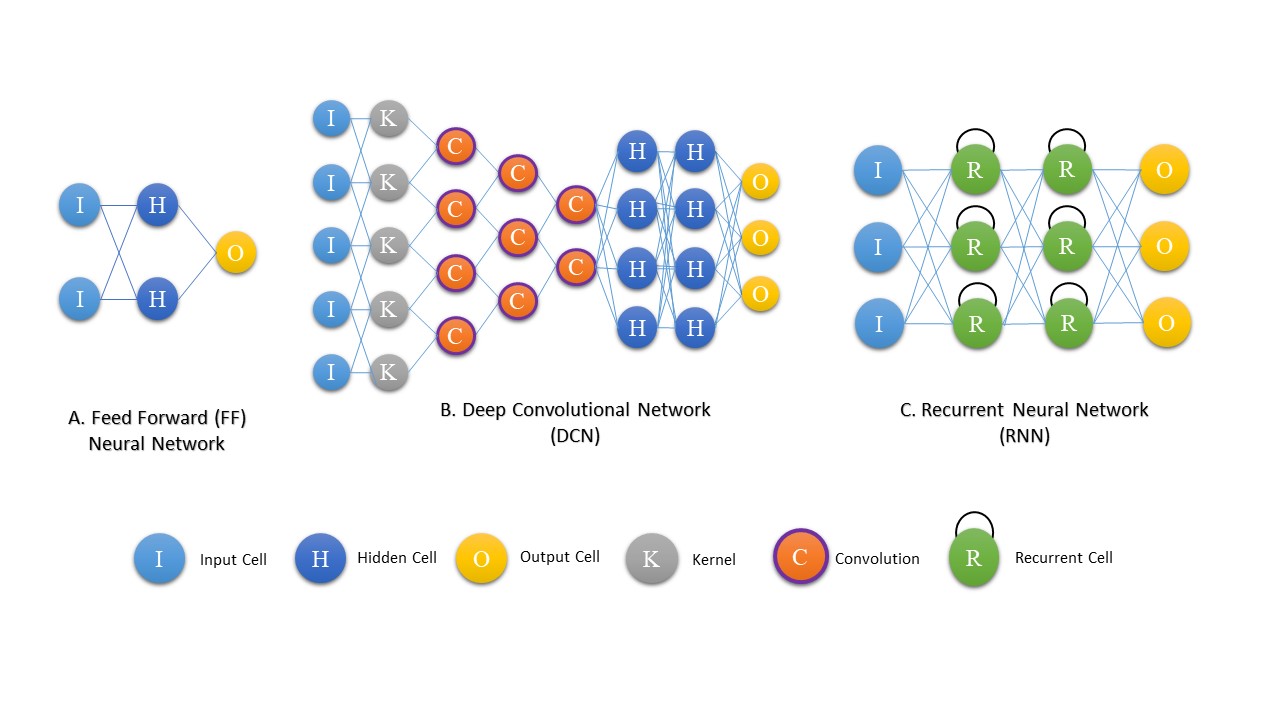

Supplement: Supplementary file 1 [file pharmaceuticals-14-00680-s001.zip › Fig S1.jpg]

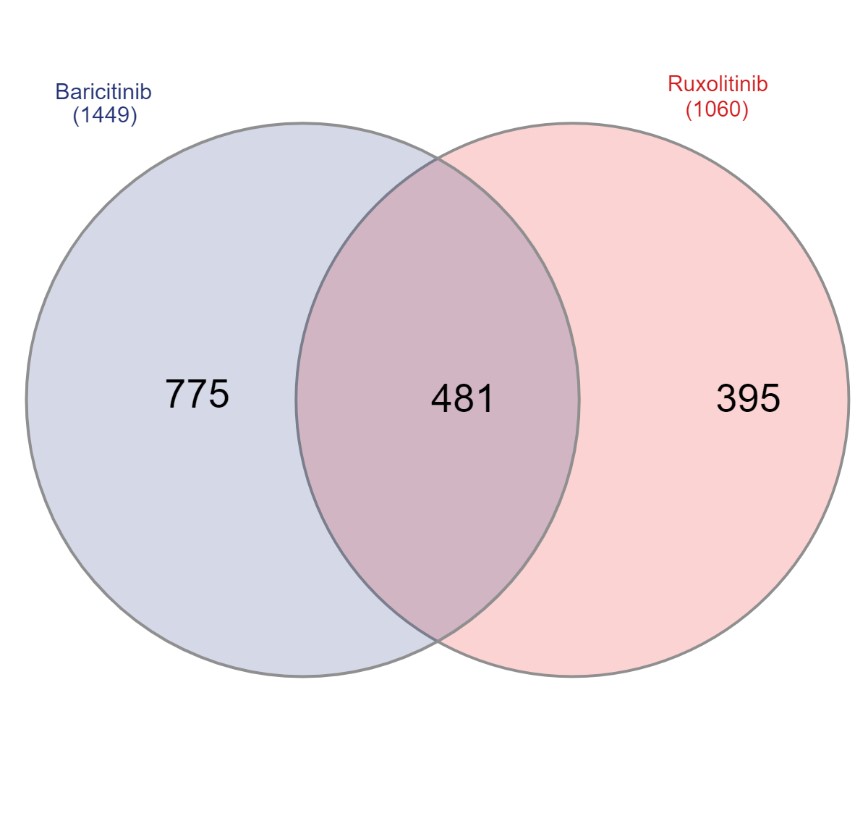

Supplement: Supplementary file 1 [file pharmaceuticals-14-00680-s001.zip › Fig S2.jpg]

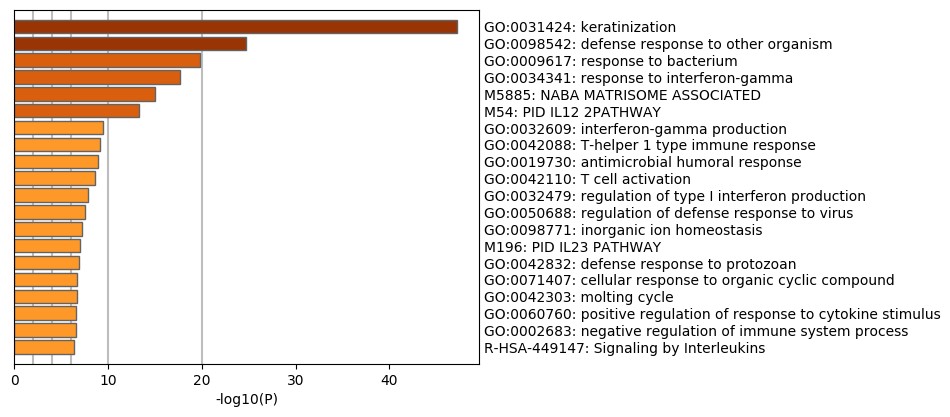

Supplement: Supplementary file 1 [file pharmaceuticals-14-00680-s001.zip › Fig S3.jpg]
